# Oral Microbiota—A New Frontier in the Pathogenesis and Management of Head and Neck Cancers

**DOI:** 10.3390/cancers14010046

**Published:** 2021-12-23

**Authors:** Marjut Metsäniitty, Shrabon Hasnat, Tuula Salo, Abdelhakim Salem

**Affiliations:** 1 Department of Oral and Maxillofacial Diseases, Clinicum, University of Helsinki, 00014 Helsinki, Finland; marjut.metsaniitty@helsinki.fi (M.M.); shrabon.hasnat@helsinki.fi (S.H.); tuula.salo@helsinki.fi (T.S.); 2 Translational Immunology Research Program (TRIMM), Research Program Unit (RPU), University of Helsinki, 00014 Helsinki, Finland; 3 Department of Pathology, Helsinki University Hospital (HUS), 00029 Helsinki, Finland; 4 Cancer and Translational Medicine Research Unit, University of Oulu, 90014 Oulu, Finland

**Keywords:** microbiome, DNA/RNA sequencing, cancer, head and neck squamous cell carcinoma, metastasis, biomarker, prognosis, treatment

## Abstract

**Simple Summary:**

Head and neck squamous cell carcinoma (HNSCC) is a group of common and aggressive tumors. Recently, oral microbiota has been credited as an important player in carcinogenesis. However, the available knowledge is not always consistent and sometimes conflicting. Therefore, the present comprehensive systematic review of the current clinical reports was conducted to evaluate the role of oral microbiota in HNSCC. Importantly, this study addresses whether oral microbiota targeting could provide diagnostic, prognostic or therapeutic utility in cancer patients. We also discussed the current limitations of this newly emerging field and the potential related strategies for the management of patients with HNSCC and possibly other solid tumors.

**Abstract:**

Head and neck squamous cell carcinoma (HNSCC) comprises the majority of tumors in head and neck tissues. The prognosis of HNSCC has not significantly improved for decades, signifying the need for new diagnostic and therapeutic targets. Recent evidence suggests that oral microbiota is associated with carcinogenesis. Thus, we conducted a comprehensive systematic review to evaluate the current evidence regarding the role of oral microbiota in HNSCC and whether their targeting may confer diagnostic, prognostic or therapeutic utility. Following the screening of 233 publications retrieved from multiple databases, 34 eligible studies comprising 2469 patients were compiled and critically appraised. Importantly, many oral pathogens, such as *Porphyromonas gingivalis* and *Fusobacterium nucleatum* were linked to certain oral potentially malignant lesions and various types of HNSCC. Furthermore, we summarized the association between the expression profiles of different oral bacterial species and their tumorigenic and prognostic effects in cancer patients. We also discussed the current limitations of this newly emerging area and the potential microbiota-related strategies for preventing and treating HNSCC. Whilst many clinical studies are underway to unravel the role of oral microbiota in cancer, the limited available data and experimental approaches reflect the newness of this promising yet challenging field.

## 1. Introduction

Head and neck squamous cell carcinoma (HNSCC) arises from the mucosal lining of the oral cavity, pharynx and larynx and it comprises the majority of tumors in the head and neck region [1,2]. Globally, HNSCCs are among the most prevalent cancers with an estimated incidence of 880,000 new cases and 440,000 deaths in 2020 alone [3]. In India, oral squamous cell carcinoma (OSCC) is causing most of the cancer-related deaths among men [3]. The 5-year survival rate of HNSCC remains low and has not significantly improved over the past years especially for metastatic lesions [4]. Tobacco consumption, alcohol abuse and infection with human papillomavirus (HPV) in adult males are the key risk factors for developing HNSCC. However, an increasing incidence of aggressive OSCC has been reported in young female patients with no history of exposure to such traditional risk factors [5]. Thus, there is an urgent need to identify new risk factors that could provide prognostic and therapeutic targets in HNSCC.

The oral microbiome consists of up to 750 microorganisms including bacteria, archaea, protozoa, fungi, and viruses [6,7]. In the oral cavity, microbial colonies can grow on hard and soft tissues including tongue, buccal mucosa, tonsils and palate. These surfaces provide different growth conditions and therefore the biofilms can significantly differ in their composition [6]. In a healthy state there is an equilibrium between these species, where the diversity and relative proportions are stable. In dysbiosis, however, such equilibrium is disrupted and followed by a compositional shift towards proinflammatory commensals with a reduction of beneficial microbes. These microbial changes could result in long lasting inflammatory conditions such as periodontitis [8].

An amassing body of evidence supports the association between oral microbiota and cancer. Oral dysbiosis can influence tumorigenesis by suppressing the immune response, synthesizing potent mutagens (e.g., acetaldehyde), and mediating chronic pro-inflammatory conditions [8,9]. Periodontitis, in this regard, has been linked to an increased incidence and poor survival of cancer [9,10]. In addition, certain oral potentially malignant disorders (OPMDs) were associated with dysbiosis [9,11]. However, data reporting the role of oral microbiota in cancer is not always consistent. On the one hand, bacterial genotoxins, such as cytolethal distending toxins, can promote DNA damage in the host cells. Furthermore, increased levels of bacterial species including *Fusobacterium nucleatum* (*F. nucleatum*) and *Porphyromonas gingivalis* (*P. gingivalis*) were associated with colorectal and pancreatic cancers, respectively [9,11]. On the other hand, oral microbiota were shown to mediate anti-tumor effects through carcinogen inactivation [9,12]. Additionally, bacterial-derived outer membrane vesicles have immunomodulatory effects and hence were suggested as novel therapeutic agents in cancer [13,14]. Therefore, we aimed to compile and analyze the current evidence regarding the association between oral microbiota and the various aspects of carcinogenesis and their potential clinical utility in patients with HNSCC.

## 2. Materials and Methods

### 2.1. Protocol and Study Registration

The protocol of this study was designed according to the recommendations of the Preferred Reporting Items for Systematic Reviews and Meta-Analysis (PRISMA). The study was retroactively registered in the International Prospective Register of Systematic Reviews (PROSPERO; registration number CRD42021256877) prior to the initiation of the systematic search [15].

### 2.2. Inclusion and Exclusion Criteria

We included original research studies that assessed the relationship between oral microbiota and the tumorigenesis of HNSCC in human samples. The detailed inclusion and exclusion criteria are listed in the Table S1.

### 2.3. Search Strategy and Study Screening

The literature search was conducted on the 13th of June 2021 without restrictions through four electronic databases: PubMed, Web of Science, Ovid Medline, and Cochrane. The applied search terms included: ((“oral microbiota”) OR (“oral microbiome”) OR (“oral bacteria *”) OR (“oral microbial”) OR (“oral microorganism”) OR (“oral microbe”)) AND ((“head and neck squamous cell carcinoma”) OR (“head and neck neoplasms”) OR (“head and neck cancer”) OR (“head and neck squamous cell cancer”) OR (“oral cancer”) OR (“mouth neoplasms”) OR (“laryngeal neoplasms”) OR (“gingival neoplasms”) OR (“lip neoplasms”) OR (“palatal neoplasms”) OR (“tongue neoplasms”) OR (“pharyngeal neoplasms”)). Following deduplication, studies were first assessed for eligibility, and then further screened for qualitative assessment. Two reviewers (MM and SH) independently screened and assessed the literatures. Differences in the results, if any, were resolved through discussion with a third reviewer (AS).

### 2.4. Data Extraction and Study Items

Extracted data were tabulated using the online collaborative tool Google Sheets™ (Google, Menlo Park, CA, US). The following key items were extracted from the eligible studies: The 1st author’s name, publication year, title, country, total number and age of patients, sample type, oral microbiota, tumor (type, location, stage, grade), Epstein Barr virus (EBV)/HPV status, methods, antibodies (name, dilution, company, etc.), *p*-value, confidence interval (CI), hazard ratio (HR), and prognostic data whenever applicable. Data extraction was performed and verified independently by two reviewers (MM and SH).

### 2.5. Assessment of Reporting Quality and the Risk of Bias

The included prognostic studies were evaluated using items adapted from the Reporting Recommendations for Tumor Marker Prognostic Studies (REMARK) guidelines [16]. The assessment criteria are detailed in the Table S2. For analyzing the risk of bias, we used the Meta-Analysis of Statistics Assessment and Review Instrument (MAStARI) tool as we recently described [17]. The assessment was performed independently by two reviewers (MM and SH). Disparities, if any, were resolved through discussion with a third reviewer (AS).

## 3. Results

### 3.1. Study Selection

**Figure 1:**
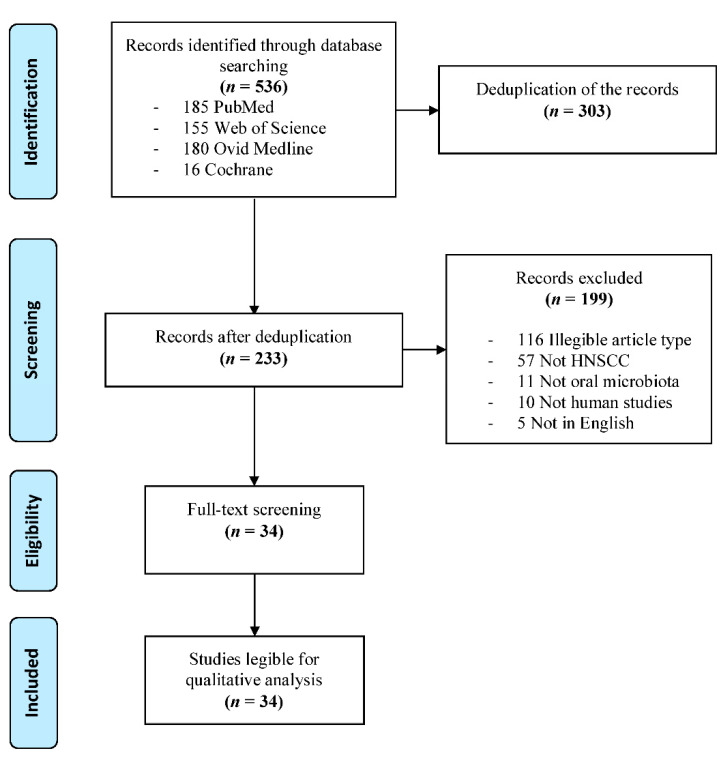
PRISMA flowchart of the study selection process.

A total of 536 studies were retrieved through the initial searches. After deduplication, 233 articles were identified and screened for eligibility. Of these, 34 studies were deemed relevant and selected for further qualitative analysis and data extraction. The search and selection process is summarized in Figure 1.

### 3.2. Baseline Characteristics of the Studies

The included 34 studies comprised a total of 4432 participants (including 2294 cancer patients, 175 OPMDs and 1963 cancer-free controls). Studies were conducted between 2005–2021 and they were based in the following countries: US (*n* = 10), Taiwan (*n* = 5), China (*n* = 5), India (*n* = 3), Japan (*n* = 2), Malaysia (*n* = 1), Australia (*n* = 1), Sri Lanka (*n* = 1), New Zealand (*n* = 1), Colombia (*n* = 1), Hong Kong (*n* = 1), Poland (*n* = 1), Brazil (*n* = 1), and France (*n* = 1). The samples were obtained from patients with HNSCC, of which OSCC was the most frequently studied tumor [18,19,20,21,22,23,24,25,26,27,28,29,30,31,32,33,34,35,36,37,38,39,40,41,42]. The studies also included samples from the following: oropharyngeal squamous cell carcinoma (OPSCC) [21,43], gingival squamous cell carcinoma (GSCC) [44]; unspecified oral cavity cancer (OCC) (45); oropharyngeal cancers (OPC) [45]; nasopharyngeal carcinoma (NPC) [46]; unspecified HNSCCs [47,48,49,50,51]. In addition, certain oral potentially malignant disorders (OPMDs), such as oral leukoplakia, were also studied. The main characteristics of the included studies are listed in Table 1.

**Figure 2:**
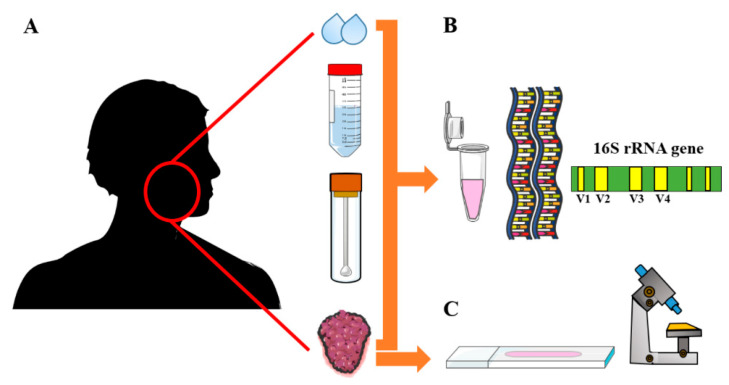
The different methods used for collecting and characterizing oral microbiota from patients with head and neck squamous cell carcinoma. (**A**) Oral microbiota samples are obtained by means of saliva expectoration; mouth wash samples; oral swab samples; or tissue biopsy. (**B**) Oral microbiota were characterized by DNA-DNA hybridization; primers targeting the 16S ribosomal RNA gene. (**C**) Immunohistochemistry of tumor sections was also used to identify oral microbiota.

The16S ribosomal RNA (rRNA) gene polymerase chain reaction (PCR) was the most commonly used approach for oral bacterial detection and identification. The sampling and characterization methods of the oral microbiota are summarized in Table 2 and Figure 2.

### 3.3. Reporting Quality and the Risk of Bias

To assess the reporting quality of the included studies, we applied six REMARK-adapted items. Of note, only one study fulfilled all of the applied criteria while the rest had at least one missing item. The MAStARI tool revealed that the risk of bias was low in 15 studies (44%), moderate in 14 studies (41%), and high in 5 studies (15%). The results for each study using the REMARK and MAStARI assessment tools are detailed in the Table S3.

### 3.4. Oral Microbiota and OPMDs

The relationship between oral microbiota and OPMDs was assessed in 6 studies. Schmidt et al. found that pyhla Firmicutes and Actinobacteria were significantly decreased in the “pre-cancer” lesions including mild, moderate and severe oral epithelial dysplasia compared to the healthy controls [41]. In agreement, Lee et al. found that the epithelial precursor lesions (e.g., dysplasia) had less abundant genera such as Bacillus, Enterococcus, Parvimonas, Peptostreptococcus and Slackia [22]. In contrast to these findings, Gopinath et al. showed that Megaspheara, unclassified Enterobacteriae, Prevotella, and Salmonella were more expressed in oral leukoplakia compared to healthy controls. Additionally, the authors reported a clear overlap between the whole mouth fluid bacteriome of leukoplakia and oral cancer [33]. Similarly, Hashimoto et al. found a significantly higher level of the genus Streptococcus in oral leukoplakia than in OSCC [30]. This was further supported by Ganly et al., who showed that Genera Fusobacterium and Veillonella were significantly increased in OPMDs [29]. Mok et al. demonstrated that phyla Firmicutes and Bacteroidetes had more OPMD related bacteria groups compared to healthy and cancer groups [42].

### 3.5. Oral Microbiota and OSCC

As the findings from OSCC studies are extensive, we will only present the statistically significant results. Recently, phyla of *Actinobacteria* and *Cyanobacteria* were decreased in OSCC (*p* = 0.021 and *p* = 0.013, respectively) compared to paracancerous tissue from the same patients [35]. Yang et al. showed that only *Fusobacterium* was increased while *Streptococcus*, *Haemophilus*, *Porphyromonas* and *Actinomyces* were all decreased (*p* < 0.0001) in OSCC compared to healthy controls [26]. In another recent study, genera *Peptostreptococcus*, *Fusobacterium*, *Alloprevotella*, and *Capnocytophaga* were all increased in contrast to decreased *Rothia* and *Haemophilus* (*p* < 0.05) in OSCC compared to non-cancer controls [31]. In agreement, OSCC patients had elevated levels of *Peptostreptococcus*, *Fusobacterium*, *Alloprevotella* and *Capnocytophaga*, while *Rothia Streptococcus* and *Veillonella* were all decreased (*p* < 0.05) [32]. Supporting these findings, genera *Prevotella*, *Fusobacterium* and *Alloprevotella* were increased in OCSCC (*p* = 0.019, *p* = 0.016 and *p* = 0.011, respectively) compared with normal control patients. Interestingly, these genera showed a trend to increase from healthy controls to OPMDs with their highest level seen in OSCC [29]. In contrast, *Streptococcus* was found decreased in OSCC [29]. Torralba et al. found a higher abundance of *Prevotella* in saliva from OSCC patients [34]. Hashimoto et al. demonstrated that phylum *Bacteroidetes* and genus *Solobacterium* had higher relative abundance in the OSCC group (*p* < 0.05) than patients with oral leukoplakia [30].

**Figure 3:**
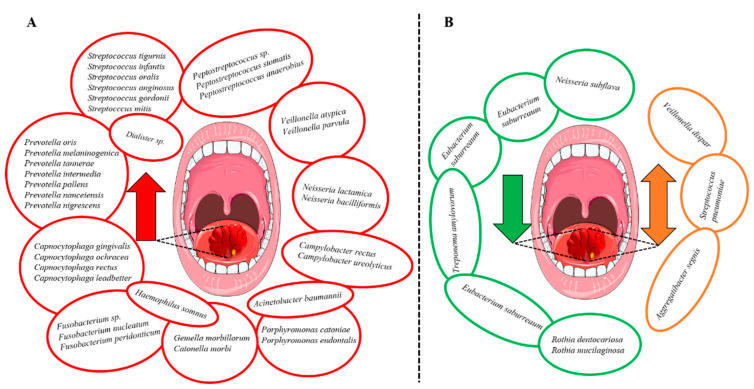
The relative abundance of the studied oral microbiota in oral squamous cell carcinoma (OSCC). (**A**) The bacterial species shown to have a higher abundance in OSCC (red circles; red arrow). (**B**) The bacterial species shown to have a lower abundance in OSCC (green circles; green arrow). In addition, a few species were shown to be increased in some studies and decreased in others (orange circles; orange double-headed arrow).

Granato et al. conducted a comparison of oral microbiota in OSCC patients before (L0) and after (L1) surgical excision [36]. Compared with the healthy controls, genera *Abiotrophia*, *Acinetobacter*, *Alloscordovia*, *Dialister*, *Gemella*, *Granulicatella*, *Peptostreptococcus*, *Selenomonas*, *Staphylococcus*, and *Stenotrophomonas* were considerably higher in both L0 and L1 patients. On the other hand, genera *Veillonella*, *Rothia*, *Moryella*, *Kingella*, and *Centipeda* were reduced in both groups. However, L1 patients had higher *Alloscordovia* and reduced *Veillonella* levels compared to L0 patients [36]. Very recently, Su et al. identified significant alterations in the bacterial diversity and relative abundance of specific oral microbiota with the most profound finding was the enrichment of *Fusobacterium* and the loss of *Streptococcus* in the OSCC [40]. Furthermore, authors suggested that genera *Streptococcus*, *Fusobacterium*, *Peptostreptococcus*, *Campylobacter* and species *Streptococcus pneumoniae* and *F. Nucleatum* (strain CTI-2) could be potential biomarkers for cancer patients [40]. The studied oral microbiota with their relative abundance in OSCC are depicted in Figure 3.

### 3.6. Oral Microbiota in Other Types of HNSCC

Debelius et al. explored the relationship between NPC and the oral microbiota using 16S rRNA sequencing of 499 NPC patients. They found that the overall microbial diversity was lower in NPC patients compared to healthy controls (*p* < 0.001). They also identified a pair of *Granulicatella adiacens* amplicon sequence variants were strongly associated with NPC status [46]. Hayes et al. studied mouthwash samples from 129 HNSCC patients including cancers of pharynx, larynx and the oral cavity. Greater levels of genera *Corynebacterium*, *Kingella*, *Neisseria*, *Abiotrophia*, *Capnocytophaga* and species *Kingella denitrificans* and *Streptococcus sanguinis* were associated with a reduced risk for larynx cancer [48]. Likewise, species *Actinomyces oris* and *Veillonella denticariosi* were associated with a reduced risk of pharynx cancer. However, there were no bacterial genera associated with oral cavity or pharynx cancer [48].

As tumor site was strongly associated with the T-stage, Wang et al. stratified their 121 samples of HNSCC based on location: oral cavity/oropharynx versus hypopharynx/larynx, phyla *Actinobacteria* was increased in low T-stage patients (*p* = 0.031), while genera *Actinomyces* and *Parvimonas* were not significantly changed [47]. In the group of oral cavity/oropharynx, *Actinobacteria* and *Actinomyces* approached significance in low T-stage patients relative to higher stages (*p* = 0.100, *p* = 0.192, respectively), while *Parvimonas* remained decreased among low T-stage patients (*p* = 0.006) [47]. In one study on GSCC, Katz et al. performed immunohistochemical staining to assess the presence of *p. gingivalis* and *Streptococcus gordonii* in tissue sections from GSCC. They showed a higher level of *P. gingivalis* (more than 33%, *p* < 0.05) in the carcinoma samples compared to normal gingiva. The staining intensity was also enhanced for *P. gingivalis* compared to specimens stained for the *S. gordonii* [44].

### 3.7. Oral Dysbiosis and Tumor Progression in HNSCC

A possible involvement of oral microbiota in dictating the progression of HNSCC was reported in seven studies [26,27,31,36,37,47,49]. For instance, *Fusobacteria* was increased during tumor development from stages 1 to 4 in patients with OSCC [26]. In contrast, *Actinobacteria* and *Bacteroidetes* were significantly decreased during cancer progression [26,47]. However, higher levels of *Bacteroidetes* have recently been linked to one of the mutational signature clusters associated with both late stages and larger sizes of OSCC [27]. Higher levels of *Fusobacterium* [49], *Rothia* [31], and *Actinomyces* [47] were associated with a lower T-stage, whereas *Parvimonas* was decreased in this stage [47] and amount of *Peptostreptococcus* positively related to a higher stage [49]. *Veillonella* was inversely correlated with clinical tumor size, lesion, and clinical stage of OSCC patients [36]. Recently, Neuzillet et al. reported that positivity of *F. nucleatum* was associated with a lower T-stage of OSCC. Furthermore, toll-like receptor 4 and the recruitment of M2-macrophages were both significantly decreased in tumors with high *F. nucleatum* load suggesting a better clinical outcome [37]. In another recent study on OSCC, Su et al. showed that oral dysbiosis can attenuate the production of anticancer metabolites such as the siderophore group nonribosomal peptides, monoterpenoid biosynthesis and others [40]. Katz et al. reported that tissue abundance of *P. gingivalis* was associated with the poorly differentiated GSCC, although it was not statistically significant [44].

The Operational Taxonomic Units (OTUs), using mothur’s Bayesian classifier, were suggested as predictors of tumor metastasis. Schmidt et al. found that 11 OTUs from *Actinomyces*, *Rothia* and *Streptococcus* were associated with OSCC while only 1 OTU from *Fusobacterium* was linked to normal samples. This set of OTUs was analyzed and it separated most cancers from other samples with the greatest separation of node metastasis cases [41]. A higher level of *Actinobacteria* was associated with TP53 mutations, while *Firmicutes* was associated with recurrent mutations in key driver genes (FAT1, FZR1, and AXIN1) related to the Wnt pathway [27]. However, Ganly et al. found no association between bacterial taxa and tumor stage or metastasis [29].

### 3.8. The Prognostic Value of Oral Microbiota in HNSCC

A total of five studies reported prognostic data associated with oral microbiota (Table 3). Recently, Granato et al. demonstrated that a higher relative abundance of *Stenophotromonas*, *Staphylococcus*, *Centipeda*, *Selenomonas*, *Alloscordovia*, and *Acinetobacter* in saliva was associated with poor overall survival of OSCC patients [36]. In contrary, *Veillonella* relative abundance inversely correlates with clinical tumor size and clinical stage, suggesting a better prognosis for OSCC patients [36]. Neuzillet et al. concluded that OSCC patients with *F. nucleatum*-positive samples had longer relapse-free survival (median: 7.06 vs. 2.11 months, *p* = 0.0091) and metastasis-free survival (9.71 vs. 3.54 months, *p* = 0.0016) compared to *F. nucleatum*-negative tumors [37]. Consistently, a higher relative abundance of *F. nucleatum* in tumor tissue was correlated with a better 3-year disease-specific survival and disease-free survival. This elevated status of *F. nucleatum* was also associated with non-smokers, lower tumor stage, lower rate of recurrence. Further, depletions of *Neisseria*, *Haemophilus*, and *Rothia* in HNSCC cases were associated with worse cancer-specific survival [49]. Robayo et al. analyzed the survival data of HPV-positive OSCC patients with those co-infected with HPV and *Streptococcus anginosus*. A tendency towards a poorer survival outcome was recognized for patients co-infected with both microorganisms, although it was not statistically significant [43]. As a novel finding, *Capnocytophaga,* a genus of gram-negative bacteria, was significantly increased in patients with recurrent OSCC after tumor resection, with a median abundance of 5.62-fold higher than in normal control patients [29].

## 4. Discussion

In the present systematic review, we summarized and analyzed a total of 34 studies involving 4432 participants, of which 2294 were HNSCC patients. The included studies evaluated the relationship between oral microbiota and HNSCC. Bacterial genera that were increased in abundance in HNSCC patients included *Fusobacterium* [26,29,31,32,40], *Peptostreptococcus* [22,31,32,40], *Alloprevotella* [29,31,32], *Capnocytophaga* [31,32,40] and *Prevotella* [29,34,40]. Additionally, the species *Prevotella melaninogenica* [18,24], *F. nucleatum* [24,32,37,40] and *Prevotella intermedia* [24,32] were increased in HNSCC (Figure 3A). In contrast, certain bacterial genera including *Streptococcus* [26,29,32,34,40], *Haemophilus* [26,31], *Rothia* [31,32,34,36] and *Veillonella* [32,34,36] were decreased (Figure 3B). However, the findings were not always consistent since *Veillonella dispar* [24,38], *Aggregatibacter segnis* [32,38] and *S. pneumoniae* [34,40] were shown to be both increased and decreased in patients with HNSCC (Figure 3B). Survival outcomes were negatively associated with the decreased abundance of *Haemophilus* and *Rothia*. In contrast, genera *Fusobacterium* and species *F. nucleatum* were associated with improved survival and lower recurrence rates.

To our knowledge, this is the first systematic review to compile and analyze an extensive amount of clinical data regarding the association between oral microbiota and different aspects of HNSCC. Although it was challenging to perform this study, yet it exhibits several strengths. Given the newness of this field and its limited experimental approaches, our comprehensive search, via multiple databases, yielded studies with a relatively large number of patients. Secondly, the study design was based on the PRISMA guidelines with a PROSPERO-registered protocol. Finally, the included studies were qualitatively appraised using both quality control and bias analysis tools. However, we acknowledge certain limitations. More than half of the eligible studies showed a tendency towards a reporting bias (i.e., moderate and high). This could be in part explained by the failure to perform a sufficient analysis of the patients’ data. Additionally, prognostic studies were limited and the REMARK criteria were not always applied. Unfortunately, variations among the studies did not permit us to conduct a meta-analysis. Finally, our analysis was restricted only to data reported in English language; thus, this study may not reveal all the available knowledge in the field.

The analyzed microbiota was isolated from saliva, oral swabs, and/or tissue samples (Figure 2). Although saliva was the most commonly used sample, there was a variation in the collection techniques including oral washes, stimulated and unstimulated saliva. These variations could subsequently affect the bacterial composition and hence the results’ compatibility [6,7]. Furthermore, results inconsistency in onco-microbiome studies could arise from focusing on the compositional analysis alone, which does not account for functional redundancy in oral microbiota [25]. Tissue samples were utilized only in a few studies with a smaller cohort size as compared to studies based on salivary analysis. Noteworthy, the controls were not always healthy or cancer-free individuals in these studies. In a recent OSCC study, for instance, the negative controls comprised patients with thyroid nodules (benign and malignant). However, these patients were considered representative of normal population as they showed no evidence of oral cavity pathology following close clinical examinations [29].

Oral microbiota were shown to be associated with cancers other than HNSCC including lung, colorectal and pancreatic cancers [9,11]. Importantly, carcinogenesis has recently been linked to periodontitis—a chronic inflammation largely mediated by oral dysbiosis [10]. In a recent meta-analysis, periodontitis and periodontal bacteria were associated with an increased incidence of cancer and poor survival rates. Interestingly, authors found that a higher cancer risk was associated with *P. gingivalis* and *P. intermedia* but not with *F. nucleatum*, *Tannerella forsythia*, *Treponema denticola* or *Aggregatibacter actinomycetemcomitans* [10]. *Fusobacterium*, specifically *F. nucleatum*, has a strong association to the tumorigenesis of colorectal cancer [52,53,54]. In agreement with this evidence, our review demonstrated that genus *Fusobacterium* [26,29,31,32,40] and *F. nucleatum* [24,32,37,40] are connected to HNSCC. However, it was also proposed that abundance of *Fusobacterium* could have a favorable effect on HNSCC progression and survival [37,49]. Another well-studied species is *P. gingivalis*, an anaerobic bacteria that has been connected among others to pancreatic cancer [55,56] and OSCC [57,58,59]. Noteworthy, only one study showed a statistically significant evidence of the association between *P. gingivalis* and HNSCC using immunostaining on tissue samples [44]. This finding raises the question whether stimulated/unstimulated saliva, swabs or tissue samples would represent the most reliable method for analyzing oral microbiota in cancer patients. Data are, however, conflicting in this regard. While unstimulated saliva was considered inferior to stimulated saliva [60,61], another study showed that there are no major differences in their reliability [62].

The utility of oral microbiota as biomarkers in HNSCC has also been elucidated. Lee et al. proposed that differences in the abundance of genera *Bacillus, Parvimonas, Peptostreptococcus* and *Slackia* could be used as a marker for the prediction, detection, and prognosis of patients with OSCC [22]. Su et al. had results supporting that the profusion of *F. nucleatum* (strain CTI-2) and a decreased abundance of *S. pneumoniae* could distinguish cancers from healthy controls [40]. Indeed, a prediction tool for metastasis would be crucial since locoregional metastasis can drastically worsen the prognosis of HNSCC patients [63]. In this context, it was suggested that certain OTUs can separate node-positive cases from the negative ones in patients with OSCC [41]. Additionally, studies indicated that oral microbiota can substantially change through the course from potentially malignant lesions to cancer [22,29,33]. This finding could have a clinical utility in future. Currently, the follow-up of OPMDs is based on the clinical appearance of the lesion and, if needed, a surgical biopsy. Thus, obtaining saliva to follow up any changes in the microbiota, together with the clinical inspection, could provide clinicians with a simple, non-invasive, approach to early diagnosing malignant changes of OSCC.

The potential role of oral microbiota in modifying the immune response in tumor microenvironment (TME) has been studied. For instance, it has been shown that infection with *F. nucleatum* enhanced M2 polarization of macrophages through TLR-4 activation, which increased tumor growth in colorectal cancer [64]. In this regard, higher levels of M2 macrophages showed poorer prognosis and clinical outcomes in HNSCC patients [65,66]. Controversially, one study reported that high levels of *F. nucleatum* was associated with decreased recruitment of M2-macrophages and low TLR4 signaling and lower T-stage [37]. Patients with HNSCC are often given antibiotics during the course of treatment. In the included studies, the oral microbiota samples were collected either prior to the treatment or without reporting which antibiotics were given. Thus, a detailed follow-up regarding the change in oral microbiota due to the administration of antibiotics and their possible effect on cancer progression is needed in the future studies.

## 5. Conclusions

This systematic review asserts the association of oral microbiota to the tumorigenesis of HNSCC. Microbial dysbiosis is evident in HNSCC patients and several bacterial genera and species seem to affect HNSCC progression, metastasis, recurrence, and/or survival. However, it remains uncertain exactly which genera, species or bacteria combinations are truly significant. Therefore, we encourage further research in this newly emerging area, which could lead to the development of effective diagnostic and prognostic targets and even therapeutic measures for patients with HNSCC.

## Figures and Tables

**Table 1:** The baseline characteristics of the included studies.

Study	Study Origin	Lesion Type(s)	Lesion Site(s)	Tumor Stage/Grade	Number of Lesion Cases	Age (Years)	Gender M/F	Study Period
[18]	US	OSCC	Oral cavity	-	45	57.6 (±2.34) (M)	32/13	-
[44]	US	GSCC	Gingiva	-	10	-	-	-
[19]	US	OSCC	Floor of the mouth	-	3	>50	3/0	-
[20]	US	OSCC	Tongue, floor of the mouth	T1–T4b, N0–N2b	10	59 (Med)	†.	-
[41]	US	OSCC, OPMDs	Buccal mucosa, tongue, gingiva, alveolar ridge, floor of the mouth, retromolar trigone	Study 1: pT2–pT4, N0–N2b; Study 2: CIS, T1–T4b, N0–N2b	Study 1: 5; Study 2: 16; OPMDs: 8	Study 1: 69.2; Study 2: 63.37; OPMDs: 58.5 (M)	18/11	2011–2012
[21]	US	OSCC, OPSCC	Oral cavity, oropharynx	-	121	58 (Med)	94/27	2011–2013
[22]	Taiwan	OSCC, OPMDs	Tongue, floor of the mouth, lip, buccal mucosa, alveolar ridge, hard palate	I–IV	OSCC: 125; OPMDs: 124	OSCC: 53 ± 10; OPMDs: 50 ± 11 (M)	223/26	2014–2015
[42]	Malaysia	OSCC, OPMDs	Oral cavity	-	18 (9 per group)	OSCC: 60; OPMDs: 54 (M)	6/12	-
[47]	US	HNSCC	Oral cavity, oropharynx, hypopharynx, larynx	I–IV	121	63 ± 11 (M)	74/47	2003–2014
[23]	China	OSCC	Oral cavity	-	40	62 (Med)	24/16	-
[48]	US	HNSCC	Oral cavity, pharynx, larynx	-	129	Group 1: 71; 2: 62.7 (M)	100/29	1992–2010
[24]	Taiwan	OSCC	Oral cavity	-	138	54.7 ± 1.2; 53.4 ± 1.3 (M)	-	2010–2013
[45]	Australia	OCC, OPC	Oral cavity, oropharynx	I–IV	52	65 (M)	46/6	-
[25]	Sri Lanka	OSCC	Tongue, buccal mucosa	Well/moderately diff.	25	61.00 ± 9.5 (M)	25/0	-
[50]	New Zealand	HNSCC	Oral cavity, left parotid, tonsils	-	14	49–81 (Range)	11/3	-
[26]	Taiwan	OSCC	Tongue, gingiva, floor of the mouth	I–IV	197	32–87 (Range)	177/20	-
[27]	Taiwan	OSCC	Buccal mucosa, tongue, lip, gingiva, others	I–IV	39	53.33 ± 10.95 (M)	39/0	2014–2015
[28]	US	OSCC	-	Non-metastatic OSCC	4	40–64 (Range)	4/0	-
[29]	US	OSCC, OPMDs	Oral cavity	-	OSCC: 18; OPMDs: 8	OSCC: 59.8 ± 10.9 OPMDs: 66.1 ± 17.9 (M)	16/10	-
[30]	Japan	OSCC, OPMDs	Tongue, gingiva, buccal mucosa	CIS, I–IV	12 (6 per group)	OSCC: 50.66; OPMDs: 58.33 (M)	9/3	-
[43]	Colombia	OPSCC	Oropharynx	-	26	31 ≥ 70 (Range)	17/9	2014–2017
[31]	Japan	OSCC	Oral cavity	T1–4, N0–3	60	63.7 (M)	50/10	2016–2018
[32]	China	OSCC	Buccal mucosa	I–IV	50	60.7 (M); 61 (Med)	32/18	2018
[49]	Hong Kong	HNSCC	Oral cavity, oropharynx, larynx and others	T1–4, N0–2	68	>60 y = 48, ≤60 y = 20	51/17	2015–2018
[46]	China	NPC	Nasopharynx	-	499	48.4 (M)	356/143	2010–2014
[33]	India	OSCC, OPMDs	Floor of the mouth, buccal mucosa, tongue, gingiva	Well/moderately/poorly diff.; Lymph node status (+/−)	OSCC: 31; OPMDs: 20	OSCC: 49.31 ± 13.24 OPMDs: 45.67 ± 6.81 (M)	-	-
[34]	Poland	OSCC	Tonsil, throat, floor of the mouth, tongue	-	18	-	-	-
[35]	China	OSCC	Oral cavity	I–IV	24	61.1 ± 12.4 (M)	17/7	-
[51]	China	HNSCC	Larynx, hypopharynx, other	I–IV	56	61.5 ± 8.8 (M)	56/0	-
[36]	Brazil	OSCC	Oral cavity (non-active lesion: L0; active L1)	T1–4; Lymph node status (+/−)	16 (8 per group)	L0: 55.8; L1: 57.7 (M)	14/2	-
[37]	France	OSCC	Oral cavity	I–IV	151	57 (Med)	93/58	1990–2006
[38]	India	OSCC	-	T2–4, N0–3; Well/mod diff.	25	55.32 (M)	16/9	-
[39]	India	OSCC	-	Well diff.	50	52.68 (M)	32/18	-
[40]	Taiwan	OSCC	Buccal mucosa	I–IV	116	54.81 ± 10.73	116/0	-

† The authors stated that the samples (*n* = 10) consisted of 53% male and 47% female. CIS, carcinoma in situ; Diff., differentiated; M, mean; Med, median; M/F, male/female; *N*, lymph node status; T, tumor size; pT, pathological tumor size; HNSCC, head and neck squamous cell carcinoma; NPC, nasopharyngeal cancer; OCC, oral cavity cancer; OPC, oropharyngeal cancer; OPMDs, oral potentially malignant disorders; OSCC, oral squamous cell carcinoma.

**Table 2:** The various methods applied in the included studies.

Study	Method	Sampling Type	Number of Samples	Microbiota Type	Microbiota Characterization
[18]	DNA-DNA hybridization	Whole unstimulated saliva through expectoration	274 (229 OSCC-free controls; 45 OSCC)	40 common oral bacteria were tested	Digoxigenin-labeled DNA using random primer technique was used
[44]	IHC	Tissue biopsy, PEFF	15 (5 normal tissue; 10 GSCC)	*P. gingivalis*; *S. gordonii*	Rabbit polyclonal antibodies (1:1000)
[19]	16S rRNA PCR	Stimulated saliva	5 (2 matched non-OSCC controls; 3 OSCC)	Total bacterial diversity and relative abundance	PCR primers were based on the V4–V5 hypervariable region
[20]	16S rRNA PCR	DNA extraction from tissue biopsy samples	20 (10 tumor-free tissues from OSCC patients; 10 OSCC)	Total bacterial diversity and relative abundance	PCR primers for V4–V5 hypervariable region; the eubacterial primers: prbac1 and prbac2
[41]	16S rRNA PCR	Swab samples from normal controls and lesions	83 (49 normal controls; 34 OSCC/OPMDs)	Total bacterial diversity and relative abundance	16S rDNA V4 hypervariable region were sequenced using the Illumina MiSeq platform
[21]	16S rRNA PCR	Oral rinse samples	363 (242 normal controls; 121 OSCC/OPSCC cases)	Total bacterial diversity and relative abundance	The Illumina MiSeq primers targeting the V4 variable region
[22]	16S rRNA PCR	Unstimulated saliva	376 (127 normal controls; 124 OPMDs; 125 OSCC)	Total bacterial diversity and relative abundance	The PCR primer pair (F515/ R806) targeting the V4 region of bacterial 16S rDNA
[42]	16S rRNA PCR	Swab samples from normal controls and lesions	27 (9 normal controls; 9 OPMDs; 9 cancer)	Total bacterial diversity and relative abundance	The primer pair D88/E94 produced near full length of 16S amplicons (targets V6–V9)
[47]	16S rRNA PCR	Paired normal and tumoral resection specimens	242 (121 tumor-free controls; 121 tumors)	Total bacterial diversity and relative abundance	PCR of the V1–V4 hypervariable regions of the 16S rRNA gene using the M13 primers
[23]	16S rRNA PCR	Swab samples from normal controls and lesions	80 (40 anatomically matched normal controls; 40 OSCC)	Total bacterial diversity and relative abundance	The PCR primer pair (515F/926R) targeting the V4–V5 regions using Illumina MiSeq tool
[48]	16S rRNA PCR	Mouth wash samples	383 (254 matched normal controls; 129 HNSCC)	Total bacterial diversity and relative abundance	The PCR primer pair (347F/803R) targeting the V3–V4 variable regions of the 16S rRNA
[24]	16S rRNA PCR	Unstimulated saliva; peripheral blood (genotyping)	289 (151 matched controls; 138 OSCC)	20 species were included for case–control comparison	The PCR primer pair (341F/926R) targeting the V3–V5 regions of the 16S rRNA
[45]	16S rRNA PCR	Oral rinse samples	83 (20 normal controls; 11 high-risk; 52 tumors)	Total bacterial diversity and relative abundance	The PCR primer pair (515F/806R) targeting the V4 variable region of the 16S rRNA
[25]	16S rRNA PCR	Tissue biopsy samples	52 (27 oral fibroepithelial polyp as controls; 25 OSCC)	Total bacterial diversity and relative abundance	The PCR primer pair (27FYMF/519R) targeting the V1-V3 regions of the 16S rRNA
[50]	16S rRNA PCR	Unstimulated whole saliva	30 (7 healthy controls; 9 dental compromised; 14 HNSCC)	Total bacterial diversity and relative abundance	The PCR primer pair (341F/806R) targeting the V3–V4 variable regions of the 16S rRNA
[26]	16S rRNA PCR	Oral rinse samples	248 (51 healthy individuals; 197 OSCC)	Total bacterial diversity and relative abundance	The PCR primer pair (16SF/16SR) targeting the V3–V4 variable regions of the 16S rRNA
[27]	16S rRNA PCR	Unstimulated saliva samples	39 (OSCC)	Total bacterial diversity and relative abundance	The PCR primers (F515/R806) targeting the V4 region of the 16S rRNA
[28]	RNA amplification	Oral swab samples	15 (4 OSCC; 11 OSCC-free sites/healthy individuals)	Active communities in tumor/tumor-free areas	Illumina adapter-specific primers were used to amplify the cDNA generated from mRNA
[29]	16S rRNA PCR	Oral rinse samples	38 (12 thyroid nodules as controls; 18 OSCC; 8 OPMDs)	Total bacterial diversity and relative abundance	The PCR primer pair (347F/803R) targeting the V3–V4 variable regions of the 16S rRNA
[30]	16S rRNA PCR	Unstimulated saliva samples	16 (4 healthy controls; 6 OSCC; 6 OPMDs)	Total bacterial diversity and relative abundance	The PCR primers (F515/R806) targeting the V4 gene region of the 16S rRNA
[43]	16S rRNA PCR	Cytobrush (control); Tissue biopsy (OPSCC)	52 (26 OPSCC; 26 controls)	*P. melanogenica*, *F. naviforme*, *S. anginosus*	Species-specific construct was designed that contained analyzed bacteria sequences
[31]	16S rRNA PCR	Stimulated saliva samples	140 (80 non-cancer controls; 60 OSCC)	Total bacterial diversity and relative abundance	PCR primers were developed for V3–V4 region of the 16S rRNA gene
[32]	16S rRNA PCR	Oral swabs from tumor and normal tissues	100 (50 from non-tumor sites; 50 tumors)	Total bacterial diversity and relative abundance	The PCR primer pair (338F/806R) targeting the V3–V4 variable regions of the 16S rRNA
[49]	16S rRNA PCR	Oral rinse samples; Tissue biopsy	272 (136 non-tumor controls; 136 tumor samples)	Total bacterial diversity and relative abundance	The PCR primer pair (341F/806R) targeting the V3–V4 variable regions of the 16S rRNA
[46]	16S rRNA PCR	Saliva samples	994 (495 healthy controls; 499 patients with NPC)	Total bacterial diversity and ASVs prevalence	The PCR primer pair (341F/805R) targeting the V3–V4 variable regions of the 16S rRNA
[33]	16S rRNA PCR	Unstimulated whole mouth fluid	74 (23 healthy controls; 31 OSCC; 20 OPMDs)	Total bacterial diversity and relative abundance	The PCR primer pair (319F/806R) targeting the V3–V4 variable regions of the 16S rRNA
[34]	16S rRNA PCR	Saliva samples; Tissue biopsy	59 (18 non-tumor tissues;18 tumor tissue; 23 OSCC saliva)	Total bacterial diversity and relative abundance	Adaptor-ligated 16S primers targeting the V4 region of the 16S rRNA gene fragment
[35]	16S rRNA PCR	Tissue biopsy samples	48 (24 paracancerous control tissues; 24 tumor tissues)	Total bacterial diversity and relative abundance	The PCR primer pair (341F/806R) targeting the V3–V4 variable regions of the 16S rRNA
[51]	16S rRNA PCR	Unstimulated saliva samples	120 (64 healthy controls; 56 from cancer patients)	Total bacterial diversity and relative abundance	The PCR primer pair (341F/806R) targeting the V3–V4 variable regions of the 16S rRNA
[36]	16S rRNA PCR	Unstimulated saliva samples	24 (8 healthy controls; 16 OSCC)	Total bacterial diversity and relative abundance	The PCR primer pair (515F/806R) targeting the V4 region of the 16S rRNA was used
[37]	16S rRNA PCR, IHC	Tissue biopsy samples	212 (HNSCC)	*F. nucleatum*; gram-negative bacteria	A unique PCR primer for F. nucleatum; LPS monoclonal Mouse antibody (clone C8)
[38]	16S rRNA PCR	Unstimulated saliva samples	49 (24 healthy controls; 25 OSCC)	Total bacterial diversity and relative abundance	The PCR primer pair (16SF/16SR) targeting the V3–V4 variable regions of the 16S rRNA
[39]	16S rRNA PCR	Tissue biopsy samples	100 (50 paracancerous control tissues; 50 tumor tissues)	Total bacterial diversity and relative abundance	A PCR primer pair targeting the V3–V4 variable regions of the 16S rRNA was used
[40]	16S rRNA PCR	Oral swabs from tumor and normal tissues	232 (116 contralateral normal tissues, 116 tumor tissues)	Total bacterial diversity and relative abundance	The PCR primer pair (515F/806R) targeting the V4 region of the 16S rRNA was used

*F. naviforme*, *Fusobacterium naviforme*; *F. nucleatum*, *Fusobacterium nucleatum*; HNSCC, head and neck squamous cell carcinoma; IHC, immunohistochemistry; LPS, Lipopolysaccharide; NPC, nasopharyngeal cancer; OPMDs, oral potentially malignant disorders; OSCC, oral squamous cell carcinoma; *P. gingivalis*, *Porphyromonas gingivalis*; *P. melanogenica*; *Prevotella melanogenica*; PCR, polymerase chain reaction; rRNA, ribosomal RNA; *S. anginosus*, *Streptococcus anginosus; S. gordonii*, *Streptococcus gordonii*.

**Table 3:** The prognostic value of oral microbiota in head and neck squamous cell carcinoma.

Study	Cancer Type	Statistics	Analysis Target	Analysis Results	Prognostic Effect	Result Interpretation
[29]	OSCC	Kruskal Wallis and Mann–Whitney tests	RA and recurrence	*Capnocytophaga* was higher in recurrent tumors (median = 1.54 vs. 0.27%); *p* = 0.0083	Unfavorable	*Capnocytophaga* is associated with OSCC recurrence. In contrast, no taxa were associated with the tumor stage, lymph node status, or distant metastasis
[43]	OSCC	Kaplan-Meier and Log-Rank tests	HPV^+ve^ patients vs. HPV^+ve^/*S. anginosus*^+ve^ patients	No statistically significant differences were observed; *p* = 0.559	Potentially unfavorable	HPV^+ve^/*S. anginosus*^+ve^ patients tend to exhibit shorter survival outcomes although it was not significant
[49]	HNSCC	Uni-/Multi-variate analyses	RA and clinical prognostic factors	*F. nucleatum* enrichment had better 3-year DSS (86.7% vs. 47.6%, *p* ≤ 0.001); DFS (85.0% vs. 41.8%, *p* ≤ 0.001); lower T-stage	Favorable	*Fusobacterium* is an independent predictor of DSS and may facilitate the use of oral bacteria as biomarkers in patients with HNSCC
[36]	OSCC	Kaplan-Meier and Log-Rank tests; Pearson correlation coefficient and cross-tabulation with the chi-square test	RA and clinical prognostic factors	Enrichment of six genera (*Stenotrophomonas*, *Staphylococcus*, *Selenomonas*, *Centipeda*, *Alloscardovia*, and *Acinetobacter*) had shorter OS (*p* < 0.05); *Veillonella* and *Centipeda* were correlated with tumor size and clinical stage	Unfavorable	Oral microbiota and their protein abundance have potential diagnosis and prognosis value for oral cancer patients
[37]	OSCC	Kaplan-Meier and Log-Rank tests; Uni-/Multi-variate analyses; Chi-square correlation test	Intratumoral *F. nucleatum* and clinical prognostic factors	In the merged cohort, *F. nucleatum*^+ve^ tumors had better OS than negative tumors (HR: 0.51, *p* = 0.009; 5-year OS 60.5% vs. 37.7%; 10-year OS 47.9% vs. 18.8%)	Favorable	*F. nucleatum* identified a subgroup of OSCC; it was frequent in older, non-drinking patients; associated with less lymph node invasion and distant relapse. *F. nucleatum* expression showed favorable OS (independent predictor), RFS and MFS outcomes in the merged cohort

RA, relative abundance; *F. nucleatum*, *Fusobacterium nucleatum*; HNSCC, head and neck squamous cell carcinoma; OSCC, oral squamous cell carcinoma; *S. anginosus*, *Streptococcus anginosus; S. gordonii*, *Streptococcus gordonii*; DSS, disease-specific survival; DFS, disease-free survival; OS, overall survival; MFS, metastasis-free survival; RFS, relapse-free survival.

## Supplementary Materials

The following are available online at https://www.mdpi.com/article/10.3390/cancers14010046/s1, Table S1: The inclusion and exclusion criteria applied in this study, Table S2: Evaluation criteria used to assess the reporting quality of the included studies, Table S3: The risk of bias and reporting quality of the included studies.
