# Supplementary material for: Oral Microbiota—A New Frontier in the Pathogenesis and Management of Head and Neck Cancers
Source: Cancers (Basel). 2021 Dec 23;14(1):46. doi: 10.3390/cancers14010046 (PMC8750462; doi:10.3390/cancers14010046)
Supplement: Supplementary file 1 [file cancers-14-00046-s001.zip › cancers-1477769-supplementary.pdf]

cancers

MDPI

Supplementary Materials: Oral Microbiota—A New Frontier in the Pathogenesis and Management of Head and Neck Cancers

Marjut Metsäniitty, Shrabon Hasnat, Tuula Salo and Abdelhakim Salem

Table S1. The inclusion and exclusion criteria applied in this study.

Inclusion criteria	Exclusion criteria
• Original research studies	• The retrieved records were<br>• Reviews<br>• Case reports<br>• Letters<br>• Conference proceedings
• Studies must be fully in English	• Studies in other languages
• Studies are performed on humans	• Studies not including human samples
• Studies on patients diagnosed with head and neck squamous cell carcinoma	• Studies with other cancer types
• Studies on oral microbiota	• Studies on non-oral microbiota

Table S2. Evaluation criteria used to assess the reporting quality of the included studies.

Checklist Items	Criteria †
1. Patient samples	Cohort (retrospective or prospective) study with a well-defined study population including detailed information such as the number of cases, source, study and follow-up periods. Authors explained the treatment(s) applied to the patients and clarified if all of the patients received the same treatment or not.
2. Clinical data	The clinical data were provided including gender, age, clinical stage and histopathological grade of the tumour.
3. Immunohistochemistry	Well-described staining protocol or at least referred to original publication(s) with clear information including antibody name, dilution, company. The cutoff value of the stained area was adequately described.
4. Prognostics	The clinical endpoints of the survival analyses were precisely defined (e.g., overall survival, disease-free survival etc.).
5. Statistics	Estimated effects (HR, CI) were describing the relationship between the evaluated oral microbiota and the outcome was provided. Adequate statistical analysis (e.g., Cox regression modelling) was performed.
6. Classical prognostic factors	The relationship between the evaluated oral microbiota and other classical prognostic factors were reported.

HR, hazard ratio; CI, confidence intervals. † Adapted from the Reporting Recommendations for Tumor Marker Prognostic Studies (REMARK) guidelines.

Table S3. The risk of bias and reporting quality of the included studies.

Study	Q1.	Q2.	Q3.	Q4.	Q5	Q6.	Q7.	Q8.	Q9.	Q10.	% *	Risk of bias	Compliance to REMARK criteria
[18]	Y	Y	Y	N	N	Y	N	NA	Y	Y	66.6	Moderate	None
[44]	N	Y	Y	N	N	N	N	NA	Y	Y	44.4	High	1,3
[19]	N	Y	Y	N	N	Y	N	NA	Y	N	44.4	High	1
[20]	N	Y	Y	N	N	N	N	NA	Y	Y	44.4	High	1
[41]	Y	Y	Y	Y	N	Y	Y	NA	Y	Y	88.8	Low	1,2
[21]	Y	Y	Y	Y	Y	Y	N	NA	Y	N	77.7	Low	1
[22]	Y	Y	Y	N	N	Y	Y	NA	Y	N	66.6	Moderate	1,2

[42]	Y	Y	Y	N	N	Y	N	NA	Y	N	55.5	Moderate	None
[47]	N	Y	Y	Y	Y	Y	Y	NA	Y	Y	88.8	Low	1,2
[23]	N	Y	Y	N	N	Y	N	NA	Y	N	44.4	High	None
[48]	N	Y	Y	N	N	Y	N	NA	Y	Y	55.5	Moderate	1,5,6
[24]	Y	Y	Y	Y	Y	N	N	NA	Y	Y	77.7	Low	5
[45]	Y	Y	Y	N	N	Y	Y	NA	Y	Y	77.7	Low	1,2,6
[25]	N	Y	Y	N	N	Y	Y	NA	Y	N	55.5	Moderate	1,2
[50]	Y	Y	Y	N	N	Y	N	NA	Y	N	55.5	Moderate	1,6
[26]	N	Y	Y	N	N	Y	Y	NA	Y	Y	66.6	Moderate	1,2,5
[27]	Y	Y	Y	Y	N	Y	Y	NA	Y	N	77.7	Low	1,2
[28]	Y	Y	Y	N	N	Y	N	NA	Y	N	55.5	Moderate	None
[29]	Y	Y	Y	N	N	Y	N	NA	Y	Y	66.6	Moderate	1,4,6
[30]	Y	Y	Y	N	N	Y	Y	NA	Y	Y	77.7	Low	1,2,6
[43]	Y	Y	Y	Y	Y	Y	N	NA	Y	N	77.7	Low	1,6
[31]	Y	Y	Y	Y	Y	Y	Y	NA	Y	Y	100	Low	2
[32]	N	Y	Y	Y	Y	Y	Y	NA	Y	Y	88.8	Low	1,2,5
[49]	Y	Y	Y	Y	Y	Y	Y	NA	Y	Y	100	Low	1,2,4,6
[46]	Y	Y	Y	Y	Y	Y	N	NA	Y	Y	88.8	Low	1,5
[33]	Y	Y	Y	N	N	N	Y	NA	Y	N	55.5	Moderate	1,2
[34]	Y	Y	Y	N	N	N	N	NA	Y	N	44.4	High	1
[35]	N	Y	Y	N	N	Y	Y	NA	Y	Y	66.6	Moderate	1,2,5
[51]	Y	Y	Y	N	N	Y	Y	NA	Y	N	66.6	Moderate	1,2,5
[36]	N	Y	Y	N	N	Y	Y	NA	Y	Y	66.6	Moderate	2,4,5,6
[37]	N	Y	Y	Y	Y	Y	Y	NA	Y	Y	88.8	Low	All
[38]	Y	Y	Y	N	N	Y	Y	NA	Y	Y	77.7	Low	2
[39]	N	Y	Y	N	N	Y	Y	NA	Y	Y	66.6	Moderate	2,6
[40]	Y	Y	Y	Y	N	Y	Y	NA	Y	Y	88.8	Low	1,2,5

Y = Yes; N = No; U = Unclear; NA = Not applicable; REMARK, Reporting Recommendations for Tumor Marker Prognostic Studies. *Percentage of the (yes) responses. The assessment questions were as follows: Q1. Were there clear criteria for inclusion in the case series?; Q2. Was the condition measured in a standard, reliable way for all participants included in the case series?; Q3. Were valid methods used for identification of the condition for all participants included in the case series?; Q4. Did the case series have consecutive inclusion of participants?; Q5. Did the case series have complete inclusion of participants?; Q6. Was there clear reporting of the demographics of the participants in the study?; Q7. Was there clear reporting of clinical information of the participants?; Q8. Were the outcomes or follow-up results of cases clearly reported?; Q9. Was there clear reporting of the presenting site(s)/clinic(s) demographic information?; Q10. Was the statistical analysis appropriate?
